# Initial experience of 3-dimensional exoscope in decompression of massive lumbar disc herniation

**DOI:** 10.1186/s12893-024-02321-6

**Published:** 2024-01-24

**Authors:** Fanglong Song, Zhiqiang Zhou, Xiaozhong Zhou, Haowei Wu, Bingchen Shan, Zhentao Zhou, Jun Dai, Fengxian Jiang

**Affiliations:** Department of Orthopedic Surgery, The Second Affiliated Hospital of Soochow University, 1055 Sanxiang Road, Gusu District, Suzhou, 215004 Jiangsu China

**Keywords:** Decompression, Degenerative disc disease, Massive lumbar disc herniation, Minimally invasive surgery, Three-dimensional exoscope

## Abstract

**Objectives:**

To investigate the effect of a three-dimensional (3D) exoscope for decompression of single-segment massive lumbar disc herniation (LDH).

**Methods:**

The study included 56 consecutive patients with single segment massive LDH who underwent decompression assisted by a 3D exoscope from October 2019 to October 2022 at a university hospital. The analysis was based on comparison of perioperative metrics including decompression time, estimated blood loss (EBL) during decompression and postoperative length of stay (PLS); clinical outcomes including assessment using the visual analogue scale (VAS) and the Oswestry disability index (ODI); and incidence of reoperation and complications.

**Results:**

The mean decompression time was 28.35 ± 8.93 min (lumbar interbody fusion (LIF)) and 15.50 ± 5.84 min (fenestration discectomy (LOVE surgery)), the mean EBL during decompression was 42.65 ± 12.42 ml (LIF) and 24.32 ± 8.61 ml (LOVE surgery), and the mean PLS was 4.56 ± 0.82 days (LIF) and 2.00 ± 0.65 days (LOVE surgery). There were no complications such as cerebrospinal fluid leakage, nerve root injury and epidural hematoma. All patients who underwent decompression assisted by a 3D exoscope were followed up for 6 months. At the last follow-up, the VAS and ODI scores were significantly improved from the preoperative period to the last follow-up (*P* < 0.05).

**Conclusions:**

A 3D exoscope provides a visually detailed, deep and clear surgical field, which makes decompression safer and more effective and reduces short-term complications. A 3D exoscope may be a good assistance tool during decompression for single-segment massive LDH.

## Introduction

Low back pain (LBP) is one of the top three causes of disability in developed countries, which severely affects the quality of patients’ life and results in huge medical costs [1]. It is well known that lumbar disc herniation (LDH) is one of the most common conditions that cause LBP. Although the symptoms are not proportional to the size of disc prolapse, massive LDH is often a concern for clinicians [2]. According to a general consensus, massive LDH is defined as a condition when the herniated disc material occupies 50% or more of the anteroposterior diameter of the spinal canal as observed on magnetic resonance imaging (MRI) [3–8]. Patients with massive LDH often experience lower limb pain, paresthesia in the dermatomal lesion corresponding to the sites of their lesion, lower extremity weakness or gait instability, and even cauda equina syndrome (CES), which severely affect the quality of their daily life [5, 9].

Because of the concern related to CES, which may result from severe compression of the dural sac by massive LDH, clinicians often tend to suggest surgery to treat patients with corresponding symptoms [2, 5]. If the massive herniated intervertebral disc does not adhere to the dural sac during the operation, surgical resection is relatively safe. However, once the disc adheres to the surrounding tissue or/and calcification of the herniated nucleus pulposus exists, there may be a higher risk of occurrence of postoperative complications such as nerve root and/or dural injury, lower limb paralysis, or intracranial infection. Incidental durotomy is also the most common intraoperative complication of a spine surgery. The incidence of incidental durotomy in an open spine surgery ranges from 1 to 9% [10, 11] and it ultimately affects the outcome of spine surgeries and patients’ quality of life postoperatively [12].

In order to decrease the risk of adverse events and improve patient outcomes, optical equipment is needed to obtain an excellent visualization of the narrow operative corridor during the surgical decompression process. According to a general consensus, the curative effect of microscope-assisted spinal surgery is better than that of direct vision surgery [13]. As a widely used intraoperative amplification equipment for spinal applications, binocular microscope has been reported with notable limitations due to the lack of wide-field enhanced depth and stereopsis [14]. To resolve this issue, a three-dimensional (3D) exoscope was developed recently. Khalessi et al. reviewed 18 microneurosurgery cases, who operated with the assistance of a 3D exoscope and reported excellent optical results, including 4 aneurysms, 1 Chiari malformation, 1 anterior cervical discectomy, 2 lumbar laminectomies [15]. Kwan et al. also reported excellent surgical and clinical outcomes without any complications in 10 patients who underwent spine surgeries using a 3D exoscope, including 4 anterior cervical discectomy and fusion, 3 cervical laminectomies and 2 lumbar laminectomies, and the authors concluded that a 3D exoscope is feasible for spine surgeries [16]. Massive LDH is a spinal disorder with relative high incidence of intraoperative complications and with high requirements for subtle manipulation and amplification equipment and we speculate that a 3D exoscope can make the surgical field clearer and the operation safer when treating massive LDH, thus reducing complications and improving clinical efficacy. However, to date, few studies have reported on the use of a 3D exoscope for decompression of massive LDH. Therefore, the main objective of this study was to evaluate clinical efficacy of 3D exoscope assisted decompression.

## Methods

### Patient population

**Fig. 1:**
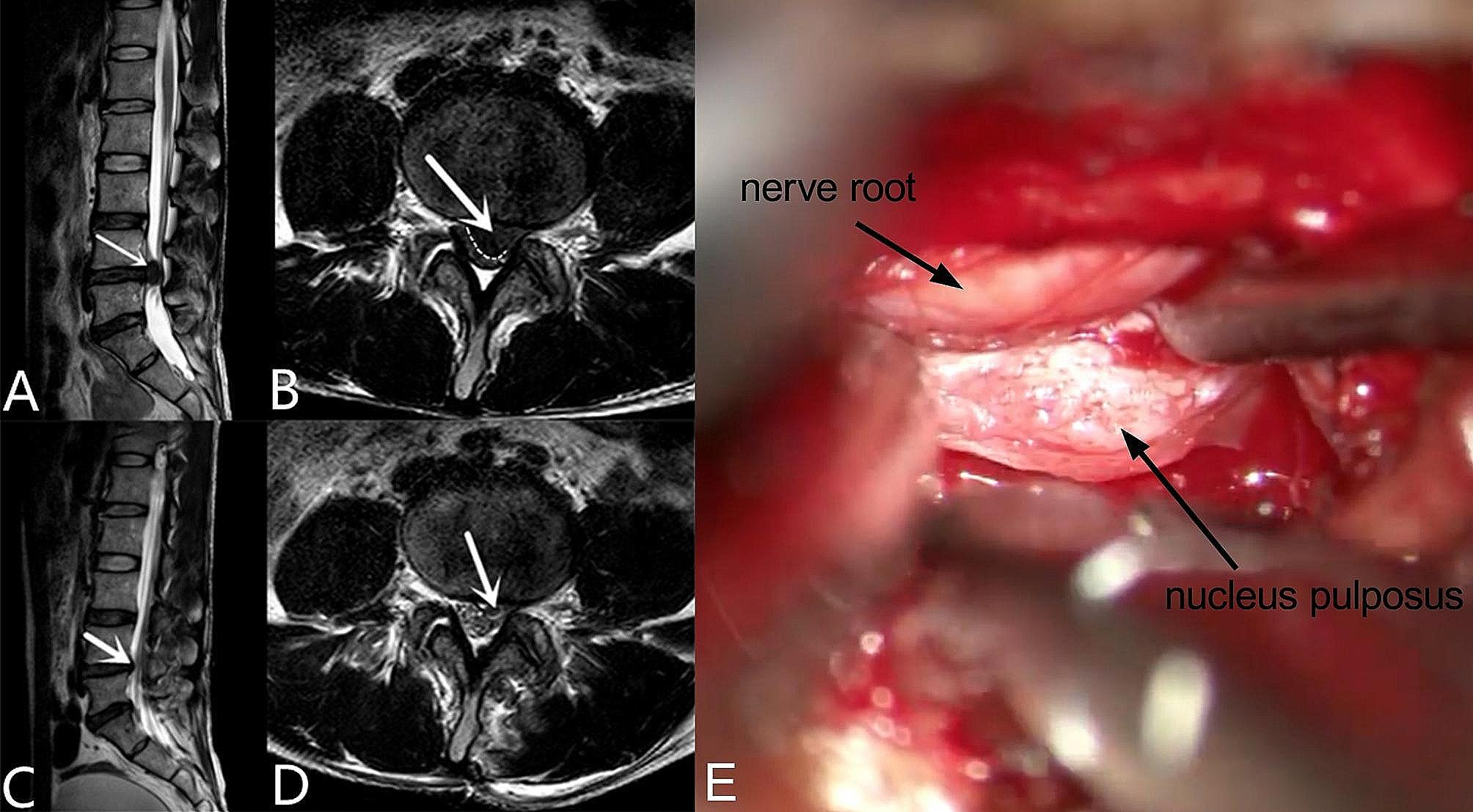
Single segment LDH observed on MRI and during surgery. A-B, massive LDH on MRI; C-D, relative small LDH; E, massive disc prolapse observed during surgery and managed with discectomy

The present study was approved by the Institutional Review Board of the Second Affiliated Hospital of Soochow University. 56 patients with single-segment massive LDH who underwent 3D exoscope-assisted decompression from October 2019 to October 2022 were retrospectively reviewed. The inclusion criteria were as follows: patients with back pain or radiating back pain related to LDH; patients with a disease course of more than 3 months, with aggravated symptoms or repeated occurrence of symptoms during the course of conservative treatment; and patients showing correlation between MRI findings and symptoms, and in whom the herniated material was found to occupy 50% of the spinal canal (Fig. 1A, B, C, D); patients with single-segment massive LDH that had developed CES which emergency surgery was required. The exclusion criteria were as follows: patients with developmental spinal stenosis, hypertrophy of ligamentum flavum, scoliosis and numerous osteophytes; severe mental illness or basic diseases; previous spinal surgeries; hemorrhagic diseases; abnormal blood coagulation and history of cancer as well as those unwilling to or unable to participate in follow-up. Patient’s termination criteria were as follows: patients who developed other unrelated diseases or major injuries during the follow-up period. Finally, 56 patients met the eligibility criteria.

Patient demographics (age, gender, comorbidities, body mass index, symptoms, and lesion location) and surgical data (decompression time, estimated blood loss (EBL) during decompression and postoperative length of stay (PLS) were collected. Clinical outcomes were assessed by the visual analogue scale (VAS) and the Oswestry disability index (ODI) preoperatively and at 1-week, 1-month, 3-month and 6-month postoperative follow-ups.

### Indications for LIF surgery

Massive LDH with segmental instability (flexion-extension radiographs taken in the standing position indicated sagittal translation of lumbar vertebral body > 3 mm or change of segmental angulation ≥ 15, or posterior opening on flexion radiograph ≥ 5) [17]. For remaining cases, LOVE surgery was performed.

### Surgical techniques

A posterior midline incision of approximately 2-3 cm (4-5 cm for LIF surgery) was made over the lumbar spine, the fascia to the side of herniation was incised along the spinous process, and the muscle was stripped subperiosteally to expose the desired lamina. After proper exposure with placement of retractors and setup and debugging of the 3D exoscope (Mitaka Kohki Co., Ltd.), A: For the LOVE surgery, overhanging of caudal lip of rostral lamina was partially removed with a Kerrison rongeur, the required amount of ligamentum flavum was removed. The dural sac along with the traversing nerve root was retracted. The herniated disc was removed using small forceps (Fig. 1E). After satisfactory disc removal, the wound was irrigated properly and closed in layers. B: For the LIF surgery, laminectomy and facetectomy of required side was performed with the ultrasonic osteotome, after removing of the herniated disc using small forceps, the disc space was identified, and standard discectomy was performed. PEEK (polyetheretherketone) cage of appropriate size filled with autologous bone graft was inserted in the disc space after adequate removal of cartilaginous endplates. Procedures of placing of pedicle screws and the rod are not included in the present study.

The surgery involved the following aspects: (1) removal of the protrusions in accordance with the imaging findings; (2) complete loosening of the nerve root at the end of the operation.

**Fig. 2:**
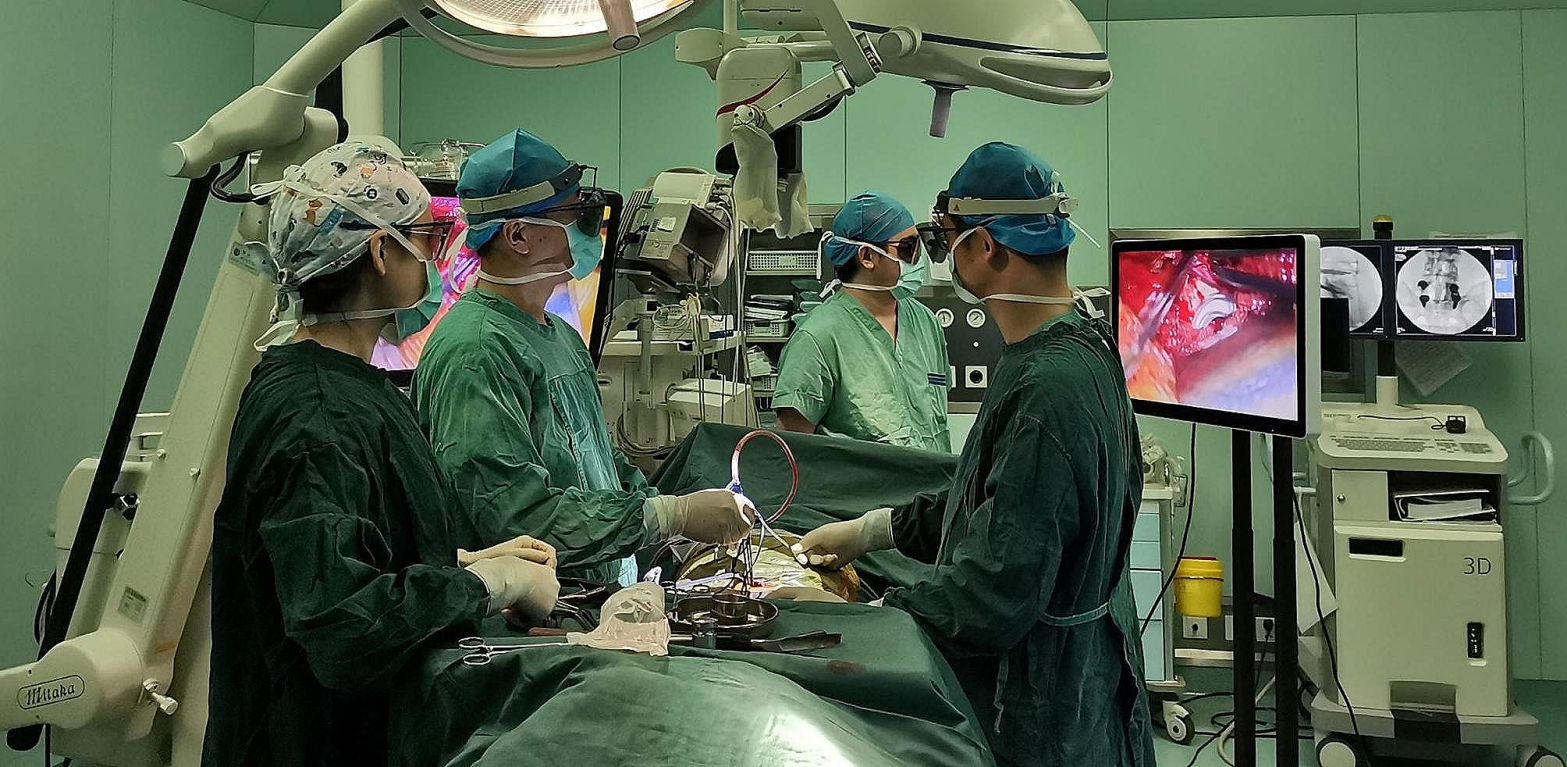
Positioning of surgeons and screens during surgery

All team members viewed the 3D monitor through the 3D glasses rather than through a binocular microscope. Both the primary and assisting surgeon shared the same operating orientation and vantage point without obstruction (Fig. 2). All the patients were given bed rest and early rehabilitation treatment after the operation and were asked to be bedridden with appropriate off-bed exercises under instructions for 2 days (LIF) or 2 weeks (the LOVE surgery) before the outpatient follow-up.

### Statistical analysis

Statistical analysis was performed using SPSS for Windows (version 22.0; SPSS, Inc., Chicago, IL, USA). Changes in periodic variables from the preoperative period to each postoperative time period were measured using the Friedman rank sum test. *P*<0.05 was considered to be statistically significant.

## Results

A total of 56 patients who underwent spinal surgery were enrolled in the present study. All patients presented with radiculopathy. 20 patients had lower extremity weakness or gait instability. 28 patients presented with paresthesia in the dermatomal lesion corresponding to the site of their lesion. Of the 56 patients, 26 patients showed involvement of the L4-L5 segment. In the remaining patients, the lesion was located in the L5-S1 segment in 24 patients and the L3-L4 segment in 5 patients (Table 1). The mean decompression time was 28.35 ± 8.93 min (LIF) and 15.50 ± 5.84 min (LOVE surgery), the mean EBL during decompression was 42.65 ± 12.42 ml (LIF) and 24.32 ± 8.61 ml (LOVE surgery), and the mean PLS was 4.56 ± 0.82 days (LIF) and 2.00 ± 0.65 days (LOVE surgery) (Table 2). Preoperative VAS score of patients with LBP and lower limb pain and ODI score were significantly improved from the preoperative period to the last follow-up after the surgery (Table 3).

None of the patients experienced postoperative headache or leakage of the cerebrospinal fluid. There were no complications such as cerebrospinal fluid leakage, nerve root injury and epidural hematoma. Revision surgery was not required in any patient during the follow-up period.

**Table 1:** Patients’ demographic data

Surgical Management	The LOVE surgery (*N* = 24)	LIF surgery (*N* = 32)
Age(yr)	32.68 ± 7.56	53.27 ± 9.40
Sex, no. (%)		
Male	13 (62.5)	16 (50.0)
Female	11 (37.5)	16 (50.0)
BMI (kg/m^2^)	25.08 ± 2.17	26.26 ± 2.33
Lesion location no. (%)		
L3-L4	2	3
L4-L5	12	14
L5-S1	9	15
Symptoms no. (%)		
Radiculopathy symptom (%)	24 (100)	32 (100)
Paresthesia (%)	10 (41.7)	18 (56.3)
Extremity weakness (%)	8 (33.3)	12 (37.5)
Comorbidities no. (%)	2 (8.3)	10 (31.3)
Cauda equina syndrome (%)	4 (16.7)	6 (18.8)

**Table 2:** Patients’ surgical data

Surgical Management	The LOVE surgery (*N* = 24)	LIF surgery (*N* = 32)
Decompression time (min)	15.50 ± 5.84	28.35 ± 8.93
EBL during decompression (ml)	24.32 ± 8.61	42.65 ± 12.42
Postoperative length of stay (d)	2.00 ± 0.65	4.56 ± 0.82

**Table 3:** Clinical outcome of patients

	Preoperation	Postoperation 1 week	Postoperation 1 month	Postoperation 3 month	Postoperation 6 month	P value
BP-VAS	6.72 ± 0.93	3.98 ± 0.38	2.59 ± 0.41	2.27 ± 0.35	1.86 ± 0.41	0.000^*^
LP-VAS	6.46 ± 0.55	2.89 ± 0.29	2.68 ± 0.34	2.32 ± 0.42	1.72 ± 0.38	0.000^*^
ODI	48.65 ± 6.62	35.85 ± 3.56	22.73 ± 3.30	17.59 ± 3.51	10.03 ± 2.23	0.000^*^

## Discussion

To the best of our knowledge, this is the first report on the evaluation of the clinical results of a 3D exoscope for decompression of massive LDH. In the present study, with the help of a clear and deep surgical field visualized by the 3D exoscope, the decompression process could be performed under the condition of minimally invasive exposure. As it was easy to operate under a 3D exoscope, there was no need for excessive traction of the patient’s incision. The internal vertebral venous plexus clearly displayed in the eyepiece is also conducive to timely hemostasis. However, in consideration of the differences in surgical techniques, habits of the surgeons and routine operations, the operation time and EBL were reported differently. Kim M et al. reviewed 748 Korean subjects and reported that the open lumbar microdiscectomy group’s average operation time was 83.99 min and the average hospital stay was 7.47 days [18]. Liu et al. reported that the mean EBL in microdiscectomy was 26 ± 15 ml [19]. Results of our present study demonstrated functional improvement with significantly improved VAS scores for back and leg pain and ODI score postoperatively. For the LOVE surgery, the mean decompression time was 15.50 ± 5.84 min, which is shorter compared with results of Kim M et al. The mean EBL during decompression was 24.32 ± 8.61 ml, which is resemble to results of Liu et al. Compared with LOVE surgery, additional procedures such as removing of cartilaginous end plates and inserting of cage for LIF led to increased decompression time (28.35 ± 8.93 min) and mean EBL (42.65 ± 12.42 ml) during the operation.

In our department, the length of stay is sometimes lengthened by some preoperative examinations such as MRI, lung function and echocardiography. However, PLS is closely related to the operation effect, complication rate and side effects. Therefore, we investigated the PLS and results showed the mean PLS was 4.56 ± 0.82 days (LIF) and 2.00 ± 0.65 days (LOVE surgery). Obviously, the PLS of LIF surgery is longer than that of LOVE surgery. We believe that this is related to the following factors: (1) Drainage tube is usually placed during LIF operation, and it cannot be removed within 2 days after surgery; (2) As a kind of internal fixation surgery, it is necessary to use antibiotics prophylactically and review the inflammation index dynamically after LIF surgery. Any signs of infection may prolong PLS; (3) Although we did not collect postoperative BP-VAS scores for LIF and LOVE patients separately and compare the data, LIF patients generally experience more implant and tissue damage-induced pain than LOVE patients, which can also prolong PLS.

Gupta A reported that complications observed in the discectomy of massive LDH were 11% [2]. Dural tear and infection are the common complications during decompression. According to a review conducted by the British Association of Spinal Surgeons, the incidence of cerebrospinal fluid leakage was 3.5% in primary discectomy and 13.2% in revision discectomy, while the infection rate was as high as 3% [20]. In most cases, the dural tear is small in size, and therefore, conservative treatment is adopted. In our study there were no complications such as cerebrospinal fluid leakage, nerve root injury or epidural hematoma. With the assistance of the 3D exoscope, the surgeon and the assistant could more accurately identify the adhesion of the nerve root, dural sac, and the adhesion between the protruding nucleus pulposus and the surrounding tissue. Intraoperative explorations were reduced, which greatly decreased the pulling time of the nerve root during decompression and accidental injury of the nerve root caused by the nerve root retractor due to the poor visual field of the assistant. Hence, less damage occurred to the dural sac and nerve root when the adhesion site was peeled off. A 3D exoscope not only makes the field of vision for this form of minimally invasive spinal surgery clearer but also enlarges it, which improves 3D spatial recognition. This enables surgeons to maneuver with precision and ease especially in high-risk patients with massive LDH or anatomical difficulties.

It is well known that patients who fail to respond to conservative treatment are treated with surgery. The aim is to remove the herniated nucleus pulposus and relieve nerve compression [21]. There are many surgical strategies for massive lumbar disc herniation, such as decompressive laminotomy; discectomy; endoscopic lumbar discectomy and transforaminal lumbar interbody fusion [2, 4, 22, 23]. There is a learning curve for any surgical procedure, but open discectomy is mastered by the vast majority of spinal surgeons. Presently, spine surgery has become minimally invasive by using a microscope or an endoscope, and effective postoperative results have been reported [24]. Chiu RG et al. concluded that patients who underwent endoscopic decompression were less likely to experience postoperative complications and surgical site infection [25]. Additionally, when the surgeons used the binocular microscope for viewing the operating field, they were required to stand higher and observe from an upward viewing angle in order to adapt to the position of the microscope oculars. A 3D exoscope negates the use of the traditional eyepiece to observe the anatomical regions and thus frees the surgeon from adopting the non-upright posture for observation, thereby relieving fatigue during long-term surgery. Joachim M. Oertel interviewed 15 surgeons in Saarland University Medical Center (Homburg-Saar, Germany) for evaluation of their intraoperative posture comfort, and all of them rated their comfort as excellent [26].

More surgical equipment and instruments will be required if interbody fusion needs to be performed in some cases; for such situations, it is necessary to optimize the operation plan and the space layout of the operating room in advance in order to avoid unnecessary prolongation of surgical time because of space congestion due to several instruments. Frykman PK believes that the exceptional intraoperative images provided by a 3D exoscope are more valuable for scrub and itinerant nurses, because these images allow them to understand better the operative process, facilitate surgical cooperation, and shorten the surgical time [27]. The 3D exoscope system allows both the surgeon and the remaining operating room team members to experience the surgical process while viewing through conveniently positioned high-definition video monitors as compared with the traditional endoscope. Our experience is consistent with the above reports.

Spinal surgery teaching often requires the adoption of the one-to-one apprenticeship mode because of small incision and narrow surgical field. A 3D exoscope allows surgeon and resident doctors to observe the surgical area from the same view interactively and immersively, thereby making the traditional, single, and dull teaching method more flexible and interesting. The video recording system of a 3D exoscope can simultaneously record the operation process, which is significantly helpful for the training of junior spinal surgeons. Thus, the use of a 3D exoscope can enable the resident doctors to observe and learn how experienced surgeons handle the instruments and to obtain better understanding of the operative process. Although quantitative assessments are lacking, resident doctors have reported to make progress in their understanding of the operative technique.

Several limitations to this study existed. As this study was designed as a preliminary application of a 3D exoscope for assistance during surgery of patients with spinal degenerative diseases, the sample size was small and the study was retrospective in nature with no control group for comparison. Further studies with a large sample size and multicenter studies are warranted to confirm the findings of the present study. All operations were performed by spinal surgeons with abundant clinical experience in our hospital, and the final conclusions may vary according to the experience of surgeons in different hospitals.

## Conclusion

In summary, a 3D exoscope provides a visually detailed, deep, and clear surgical field, which makes decompression safer and more effective, significantly reduces short-term complications, and enables resident doctors to learn effectively. However, it cannot be considered as a revolutionary or a replacement product. We believe that a 3D exoscope may be a good assistance tool during decompression for single-segment massive LDH.

## Data Availability

The datasets of the current study are available from the corresponding author upon reasonable request.
